# Steroid Hormones Are Potent and Putatively Endogenous Activators of Human Bitter Taste Receptors

**DOI:** 10.1111/nyas.70172

**Published:** 2026-01-02

**Authors:** Tatjana Lang, Francesco Ferri, Florian Ziegler, Antonella Di Pizio, Maik Behrens

**Affiliations:** ^1^ Leibniz Institute for Food Systems Biology at the Technical University of Munich Freising Germany; ^2^ Professorship for Chemoinformatics and Protein Modeling Technical University of Munich Freising Germany

**Keywords:** bitter taste receptor, endogenous agonist, functional assay, steroid hormone

## Abstract

Human bitter taste plays an important role in the quality assessment of food. The presence of the corresponding receptors, the taste receptor 2 family (TAS2Rs), in nongustatory tissues without direct contact to the environment suggested that, apart from food compounds, putative endogenous agonists may also exist. Recent studies on bitter taste receptors of vertebrates, including humans, identified occasional steroid hormones as agonists for these receptors; therefore, steroid hormones represent relevant, potentially endogenous agonists for TAS2Rs. In this study, a comprehensive analysis of 19 steroid hormones, cholesterol, and two plant‐derived hormones was performed using functional assays to assess the activation of TAS2Rs. Two TAS2Rs, TAS2R14 and TAS2R46, were found to be differentially activated by the test compounds, with TAS2R46 being in almost all cases the more sensitive receptor. Some steroid hormones activated TAS2R46 with extraordinarily high potencies. Comparison with a human metabolite database revealed that several steroid hormone levels reach activating concentrations for TAS2Rs, suggesting that TAS2Rs indeed could act as sensors for circulating steroid hormones.

## Graphical abstract

Human bitter taste receptors are not only involved in sensing tastants within the oral cavity but also play crucial roles in internal tissues of the body. The current report identifies numerous structurally and functionally diverse steroid hormones as activators of the two human bitter taste receptors, TAS2R14 and TAS2R46. As endogenous agonists of bitter taste receptors, steroid hormones could play important physiological roles. [Image created in BioRender. https://BioRender.com/985s9cz]

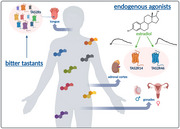

## 1 Introduction

The ability to taste bitter is thought to prevent the ingestion of noxious food compounds [1]. By far, not all bitter compounds, however, are toxic, and a moderate bitter taste is often tolerated or even appreciated by some consumers [2]. The corresponding receptors, G protein‐coupled receptors of the taste 2 receptor family (TAS2R) [3], are expressed in the oral cavity, where they occur in bitter taste receptor cells located in taste buds [4]. The number of functional bitter taste receptor genes in jawed vertebrates fluctuates considerably between one in several but not all sharks [5, 6] and 248 in anurans [7]. Some vertebrate genomes are even devoid of any functional taste receptors [8]. With about 25 intact TAS2Rs, humans possess an average‐sized bitter taste receptor gene repertoire. However, due to frequent gene deletions [9, 10] or the presence of nonfunctional genetic variants [11, 12, 13, 14], as well as the expression of rare functional TAS2Rs, such as in the case of TAS2R2 [15, 16], the number of intact TAS2R genes can fluctuate somewhat. The existence of quite broadly tuned bitter taste receptors in most species investigated so far prevents a direct correlation between the number of functional bitter taste receptors and the importance of bitter recognition for these animals [17].

The expression of taste receptors, including bitter taste receptors, is not limited to the oral cavity, as many additional tissues have been shown to express these receptors as well (for recent reviews, see Refs. [18, 19, 20, 21]. In agreement with their assumed protective role in the taste system, the majority of functions implicated for bitter taste receptors expressed outside the gustatory tissue are related to defensive activities, such as the protection of respiratory epithelia against noxious stimuli [22, 23, 24] or the secretion of antimicrobial molecules in gastrointestinal epithelia [25]. However, gastrointestinal bitter taste receptors have also been demonstrated to contribute to regulatory mechanisms in metabolic processes [26, 27, 28, 29]. As some of the extra‐oral tissues expressing bitter taste receptors, such as the brain [30, 31], heart [32], or thyroid gland [33], have no access to the outside world, the potential existence of endogenous agonists has been hypothesized [34]. Indeed, bile acids have been identified recently as agonists of human and mouse bitter taste receptors, making this group of compounds candidates for endogenously synthesized TAS2R agonists, in particular, since their determined response thresholds match circulating bile acid levels in human blood [35]. Moreover, bile acid sensitivities of bitter taste receptors are found throughout all investigated jawed vertebrates, ranging from sharks [5], bony fish [36], amphibians, birds, and rodents to humans [37], indicating pronounced phylogenetic conservation.

Bile acids share the same scaffold structure as steroid hormones [38, 39]. Steroid hormones are widely produced and highly conserved in jawed vertebrates and, although only small subsets were tested rather sporadically, were shown to activate bitter taste receptors of shark (progesterone cf. Ref. [5]), bony fish (androsterone and progesterone cf. Ref. [36]), mouse (Progesterone cf. Ref. [40]), and human (androsterone, progesterone, hydrocortisone cf. Refs. [40, 41]). In the case of human TAS2Rs, responsiveness to steroid hormones was limited to the two receptors, TAS2R14 and TAS2R46. Therefore, this compound class warrants further investigation of its bitter taste receptor activation properties. In general, steroid hormones are divided into sex steroids and corticosteroids [39, 42, 43]. Whereas sex steroids are mainly produced in the gonads, corticosteroids have their origin in the adrenal cortex [44, 45, 46]. Further known tissues of steroid hormone production are the placenta [47, 48], as well as the brain [49, 50, 51]. In general, steroid hormones interact with steroid hormone receptors (for a review, see Ref. [52]), which are ligand‐activated transcription factors. Additionally, G protein‐coupled receptors, such as the progesterone‐activated GPR30 [53], were shown to respond to steroid hormones (for a review, see Ref. [54]). The activities of sex steroids are not limited to reproductive organs [55], but they play a role in several additional physiological processes, like the increase in nitric oxide and decrease in free radical production in the case of estrogens [55, 56]. Steroid hormone plasma levels fluctuate slightly during the day as well as depending on age, but physiological concentrations are generally in pM to low nM ranges [57, 58]. Of special interest is the stage of pregnancy, when concentrations of steroid hormones like estradiol or progesterone increase enormously in human blood plasma [59].

Some typical symptoms that occur during pregnancy are morning sickness, taste disorders, especially bitter metallic taste, or low blood pressure [60, 61, 62]. Whereas the immediate increase in steroid hormones may directly influence taste receptors in the oral cavity, brain regions involved in the development of nausea as well as arteries express bitter taste receptors [63, 64]. As already mentioned before, it is already known that bitter taste receptors trigger relaxation of pulmonary arteries and, in doing so, blood pressure may be lowered [65].

To clarify the putative involvement of steroid hormones in these processes by activating bitter taste receptors, we performed a screening of all human bitter taste receptors for their activation by a set of steroid hormones. Subsequently, the two most sensitive receptors, TAS2R14 and TAS2R46, were carefully analyzed for their steroid hormone responses using an extensive set of 18 different steroid hormones, the precursor substance cholesterol, and the hormonally active plant metabolites genistein and phytosterol stigmasterol. The results were matched with known data from the Human Metabolome Database to interrogate putative physiological functions of bitter taste receptor activation by steroid hormones [66].

## 2 Materials and Methods

### 2.1 Steroid Hormones

A first screening for steroid hormone responses of 26 human bitter taste receptors was done with estradiol. Detailed characterization of the steroid hormone response of the human TAS2R14 and TAS2R46 [40] was performed using a set of 18 further steroid hormones, the precursor substance cholesterol, as well as genistein and stigmasterol (cf. Table S1). Stock solutions were prepared in dimethyl sulfoxide (DMSO). To reduce cytotoxic effects of DMSO, stock solutions were diluted to a maximum DMSO concentration of 0.5% in C1 assay buffer (130 mM NaCl, 10 mM 4‐(2‐hydroxyethyl)piperazine‐1‐ethanesulfonic acid pH 7.4, 5 mM KCl, 2 mM CaCl_2_, 0.18% glucose). Maximal applied steroid hormone concentration was limited due to solubility issues or artifacts during measurement (cf. Table S1) [35, 41].

### 2.2 Screening

The screening of human bitter taste receptors was performed using transiently transfected HEK293T‐Gα16gust44 cells (a gift from J. Slack, Givaudan) [15, 35]. The cell line was cultivated in Dulbecco's modified eagle medium (DMEM) (Thermo Fisher Scientific, Darmstadt, Germany), supplemented with 10% fetal bovine serum (Sigma Aldrich, Steinheim, Germany), 1% l‐glutamine, 100 units/mL penicillin (Sigma Aldrich), and 100 µg/mL streptomycin (Sigma Aldrich), at 37°C and 5% CO_2_. The cells were seeded onto poly‐d‐lysine‐coated 96‐well plates. The next day, when cells were grown to a confluence of 40–60%, they were transiently transfected using 150 ng of bitter taste receptor cDNA in pcDNA5 FRT vector and 0.3 µL lipofectamine2000 in serum‐free DMEM per well. The empty vector was equally transfected as a negative control (mock). After 5 h, the medium was changed to supplemented DMEM. The next day, cells were loaded with Fluo‐4‐AM (Abcam, Cambridge, Great Britain) in the presence of 2.5 mM probenecid (Sigma‐Aldrich) and washed after 1 h with C1 buffer using a BioTek Cell Washer. After 30 min, cells were washed again with C1 buffer. Application of substances and measurement of fluorescence changes upon receptor activation and resulting Ca^2+^ release were done with an automated fluorescence plate reader (FLIPR^TETRA^) (Molecular Devices, San Jose, USA). A second application with 100 nM somatostatin 14 (Bachem, Bubendorf, Switzerland) served as a cell viability control.

### 2.3 Dose–Response Relationships

For measurement of dose–response relationships, the stable inducible cell lines FLP‐In T‐Rex 293‐Gα16gust44‐TAS2R14 and TAS2R46, available from previous research, were used [67, 68]. Handling of cells was identical to the screening procedure. Expression of receptors was induced for 16–18 h for TAS2R14 and 3–5 h for TAS2R46, respectively, before measurement by adding 5 µg/mL tetracycline. Calcium imaging was done as described before.

### 2.4 Data Analysis

Fluorescence signals upon calcium release of noninduced cells were subtracted from induced cells in ScreenWorks 4.2 software, and the resulting signal amplitudes were used for further calculations. Using Microsoft Excel, the fluorescence intensities were standardized to the basal fluorescence and normalized to the buffer‐only control. Final statistics were done with SigmaPlot. For the determination of threshold concentrations and half‐maximal effective concentrations (EC_50_ concentrations), three independent experiments with two technical replicates each were performed. The EC_50_ concentrations were determined by nonlinear regression using the equation *y* = (max − min) / [1 + (*x*/EC_50_) − Hillslope] + min, where *x* corresponds to the substance concentration. All calculations and diagrams were created using SigmaPlot 14.0. Student's *t*‐test was used to determine statistical significance (*p* < 0.01).

### 2.5 Molecular Modeling

Phase (Schrödinger Release 2025‐1: Phase, Schrödinger, LLC, New York, NY, 2025) was used to generate pharmacophore models based on the identified agonists for TAS2R14 and TAS2R46. To build the models, we used agonists for which EC_50_ values were determined (16 compounds for TAS2R46 and 6 compounds for TAS2R14). The models generated were set to have from three to five pharmacophoric features, to match all actives, and 50 conformers per ligand were generated. The models with the best Phase hyposcore for each receptor were selected for the analysis. The compounds used to generate the pharmacophore models were then analyzed for their putative binding modes in the TAS2R14 and TAS2R46 orthosteric binding sites. The CryoEM structures of TAS2R14 (PDB ID: 8VY7) and TAS2R46 (PDB ID: 7XP6) were prepared with the Protein Preparation Wizard plugin available in Maestro (Schrödinger Release 2025‐1). We performed docking studies of the most active ligand for each receptor (17α‐hydroxyprogesterone, adrenosterone, aldosterone, androstenedione, androsterone, dehydroepiandrosterone, dehydroepiandrosterone sulfate, deoxycorticosterone, dihydrotestosterone, epitestosterone, estradiol, estriol, hydrocortisone, methyltestosterone, progesterone, testosterone for TAS2R46 and epitestosterone, estradiol, estrone, methyltestosterone, progesterone, testosterone for TAS2R14) using Glide Standard Precision. The best‐scoring pose was then optimized with the ligand‐protein refinement tool available in Prime (Schrödinger Release 2025‐1). The obtained refined pose was then used as a reference for superimposing agonists with defined EC_50_. This was considered the binding conformation of the investigated ligands. The complexes with the TAS2R46 receptor were then generated and refined with the ligand‐protein refinement tool in Prime (Schrödinger Release 2025‐1) using default settings. Molecular Mechanics Generalized Born Surface Area (MM‐GBSA) calculations were used to estimate the free binding energy (ΔG) of each complex (Table 1). In the case of TAS2R14, the alignment of compounds with nonaromatic ring A (i.e., epitestosterone, methyltestosterone, progesterone, testosterone) to the most active compound that has an aromatic ring as ring A (i.e., estrone) did not perform well. Therefore, these compounds were aligned to the cholesterol conformation in the PDB structure (8VY7.pdb).

**TABLE 1:** Threshold concentrations (TH), EC_50_‐concentrations, and maximal signal amplitudes (E_max_) of TAS2R14 and TAS2R46 steroid hormone‐related agonists.

	#	Compound	TAS2R14				TAS2R46			
			TH (µM)	EC_50_ (µM)	E_max_	ΔG (kcal/mol)	TH (µM)	EC_50_ (µM)	E_max_	ΔG (kcal/mol)
	1	17α‐Hydroxyprogesterone	—	—	—	—	0.01	0.33 ± 0.08	0.85 ± 0.01	−53.82
	2	Adrenosterone	30	—	0.21 ± 0.04	—	0.3	2.29 ± 0.17	1.07 ± 0.03	−44.41
	3	Aldosterone	100	—	0.09 ± 0.01	—	1	14.9 ± 1.9	0.64 ± 0.05	−40.68
	4	Androstenedione	0.3	—	0.25 ± 0.02	—	0.03	0.55 ± 0.04	1.03 ± 0.11	−44.53
	5	Androsterone	1	—	0.23 ± 0.02	—	0.1	0.87 ± 0.09	0.69 ± 0.01	−40.12
	7	Cortisone	300	—	0.29 ± 0.04	—	3	—	0.74 ± 0.04	−41.27
	8	Dehydroepiandrosterone	10	—	0.23 ± 0.03	—	0.1	1.35 ± 0.19	0.88 ± 0.11	−22.28
	9	Dehydroepiandrosterone sulfate	100	—	0.07 ± 0.01	—	0.1	4.70 ± 2.25	0.25 ± 0.03	−44.80
	10	Deoxycorticosterone	10	—	0.53 ± 0.02	—	0.01	1.07 ± 0.17	1.08 ± 0.11	−46.35
	11	Dihydrotestosterone	3	—	0.23 ± 0.03	−55.41	0.03	0.78 ± 0.18	0.79 ± 0.12	−46.31
	12	Epitestosterone	3	14.7 ± 7.6	0.17 ± 0.02	−75.03	0.01	1.29 ± 0.44	0.88 ± 0.08	−43.73
	13	Estradiol	1	7.7 ± 2.0	0.27 ± 0.01	—	1	1.66 ± 0.46	0.34 ± 0.02	−43.60
	14	Estriol	30	—	0.06 ± 0.01	−74.94	1	8.02 ± 0.66	0.46 ± 0.08	—
	15	Estrone	0.1	0.92 ± 0.27	0.37 ± 0.04	—	—	—	0.06 ± 0.02	−45.89
	16	Hydrocortisone	300	—	0.19 ± 0.03	−53.91	1	11.7 ± 2.6	0.39 ± 0.04	−48.12
	17	Methyltestosterone	3	21.0 ± 1.9	0.27 ± 0.02	−60.77	0.03	1.21 ± 0.30	0.99 ± 0.08	−47.61
	18	Pregnenolone	30	—	0.07 ± 0.02	−55.41	0.3	—	0.27 ± 0.04	−46.31
	19	Progesterone	1	38.0 ± 8.8	0.32 ± 0.05	—	0.03	0.53 ± 0.10	0.77 ± 0.05	−53.82
	20	Testosterone	1	58.7 ± 5.9	0.55 ± 0.07	—	0.03	1.62 ± 0.43	0.79 ± 0.09	−44.41
	6	Cholesterol	100	—	0.17 ± 0.03		100	—	0.05 ± 0.03	
	21	Genistein	10	—	0.18 ± 0.01		—	—	—	
	22	Stigmasterol	100	—	0.15 ± 0.01		—	—	—	

*Note*: Compound categories are indicated by colors on the left‐hand side (black = steroid hormones, white = precursor, gray = plant‐derived substances with hormonal activity). MM‐GBSA ΔG values obtained from the predicted binding poses of investigated steroid hormones activating TAS2R14 and/or TAS2R46.

Abbreviations: EC_50_‐concentrations, half maximal effective concentrations; MM‐GBSA, molecular mechanics–generalized born surface area.

All binding modes of analyzed compounds, with docking scores and MM‐GBSA‐derived ΔGs, are available at: https://github.com/DiPizio‐Lab/SteroidHormones_docking/tree/main.

## 3 Results

**FIGURE 1:**
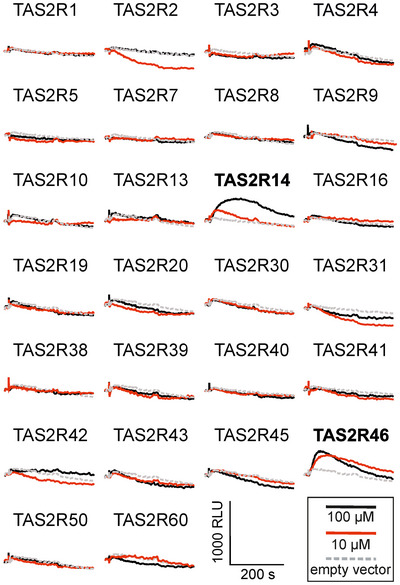
Fluorescence traces of 26 receptors screened with 10 and 100 µM estradiol.

Previous screenings of human TAS2Rs with steroid hormones revealed responses to the corticosteroid hydrocortisone, the male sex steroid androsterone, and the female sex steroid progesterone. Therefore, we opted to screen the human TAS2Rs with estradiol to check if the physiologically most powerful female sex steroid activates other TAS2Rs except for TAS2R14 and TAS2R46. The 26 receptor cDNAs were transiently transfected into HEK 293T‐Gα16gust44 cells and screened for their activation by 100 µM, the maximum artifact‐free concentration, and 10 µM estradiol. As shown in Figure 1, estradiol elicited responses in TAS2R14 and TAS2R46 transfected cells in the high and low concentrations, thus confirming that these two receptors represent the main targets for steroid hormone activation.

**FIGURE 2:**
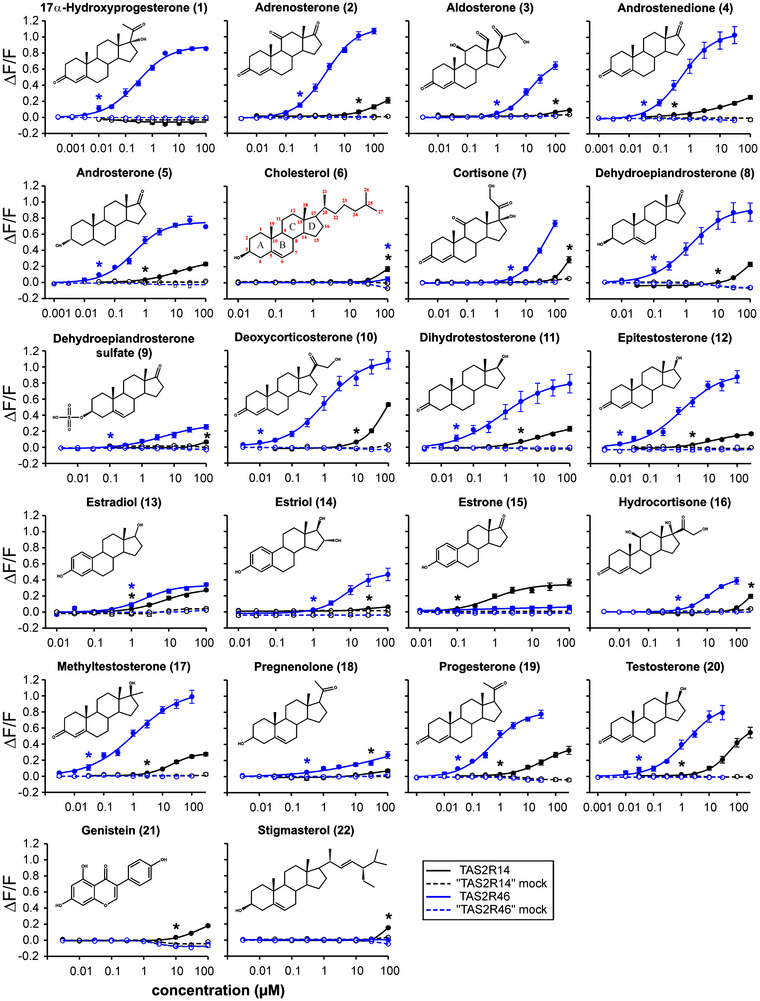
Dose−response relationships of TAS2R14 and TAS2R46 with steroid hormones and related compounds. Structural formulas of the investigated compounds are shown in the corresponding graphs. The monitoring of dose–response relationships revealed that almost all substances elicit responses with higher potencies and efficacies in cells expressing TAS2R46. The only exceptions are those substances that exclusively activate TAS2R14, namely, estrone, genistein, and stigmasterol. Asterisks indicate the lowest compound concentrations eliciting statistically significant responses (Student's *t*‐test, *p* ≤ 0.01) of receptor‐transfected cells compared to mock‐transfected cells (= threshold concentrations). The obtained threshold concentrations, EC_50_‐concentrations, and maximal signal amplitudes are summarized in Table 1.

In order to obtain a comprehensive overview of the response properties of receptors TAS2R14 and TAS2R46, we expanded the array of investigated substances to 22 and obtained dose−response relationships to determine potencies and efficacies (see Figure 2). The response patterns of the screened substances with the two TAS2Rs are summarized in Table 1. As evident from Table 1, the two receptors exhibit apparent redundancies, with TAS2R14 responding to all tested substances except for 17α‐hydroxyprogesterone and TAS2R46 being activated by most substances except estrone and the two plant metabolites genistein and stigmasterol.

The data summarized in Table 1 demonstrate the superior sensitivity of TAS2R46 compared to TAS2R14 for most tested substances. Intriguingly, the threshold concentrations of 17α‐hydroxyprogesterone, deoxycorticosterone, and epitestosterone with 0.01 µM are by far the lowest concentrations ever found to activate a human TAS2R. Another set of five substances, androstenedione, dihydrotestosterone, methyltestosterone, progesterone, and testosterone, exhibited only slightly higher threshold concentrations of 0.03 µM. Further, five substances, namely, androsterone, dehydroepiandrosterone, and dehydroepiandrosterone sulfate with 0.1 µM and adrenosterone as well as pregnenolone with 0.3 µM, respectively, showed threshold concentrations in the submicromolar range. The corticosteroids aldosterone, hydrocortisone, and the female sex hormones estradiol and estriol followed with threshold concentrations of 1 µM. Whereas cortisone still elicited responses in the low micromolar range (3 µM), the precursor cholesterol activated only at high micromolar concentration (100 µM). In sharp contrast, the only submicromolar threshold concentration for the activation of TAS2R14 was determined for the female sex hormone estrone, whereas all other compounds exhibited threshold concentrations between the low and high micromolar concentration range. Hence, despite the vastly overlapping activation patterns observed for both receptors, TAS2R46 is much more sensitive. Interestingly, the exclusive agonists, 17α‐hydroxyprogesterone for the TAS2R46 and estrone for the TAS2R14, are in both cases detected with the lowest threshold concentrations, suggesting specialized nonredundant functions. The highest efficacies were found for deoxycorticosterone (ΔF/F = 1.08 ± 0.11), closely followed by adrenosterone (ΔF/F = 1.07 ± 0.03) and androstenedione (ΔF/F = 1.03 ± 0.11) in the case of TAS2R46, and for testosterone (ΔF/F = 0.55 ± 0.07) and deoxycorticosterone (ΔF/F = 0.53 ± 0.02) in the case of TAS2R14. Hence, these compounds stood out because of the signal amplitudes they generated and, therefore, likely represent full agonists.

**FIGURE 3:**
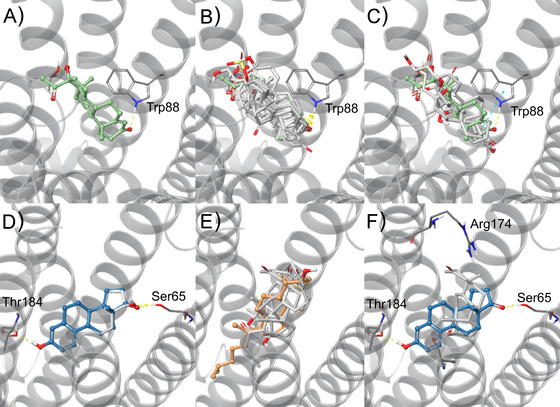
TAS2R46 (PDB ID: 7XP6) binding mode of: (A) the most active compound 17α‐hydroxyprogesterone (light green, ball&stick representation), (B) agonists with EC_50_‐concentrations lower than 5 µM (white, stick) aligned to most active (light green, ball&stick), (C) compounds with EC_50_‐concentrations higher than 5 µM (white) whose binding modes are influenced by the presence of Trp88; TAS2R14 (PDB ID: 8VY7) binding mode of: (D) estrone (blue) and estradiol (white), which are highly superimposed due to the high similarity; (E) compounds with cholesterol‐like binding mode (epitestosterone, methyltestosterone, testosterone, and progesterone, in white stick representation) together with experimental conformation of cholesterol (light orange, ball&stick) (white); and (F) comparison of the two identified binding modes (as representatives, we report estrone in blue and epitestosterone in white).

The predicted binding mode of 17α‐hydroxyprogesterone within TAS2R46 (Figure 3A) shows an anchoring hydrogen bond (H‐bond) interaction between the carbonyl oxygen on ring A and Trp88. All TAS2R46 steroid hormone agonists can assume a similar binding mode to 17α‐hydroxyprogesterone (Figure 3B), and the MM‐GBSA energy values for all compounds are in agreement with the determined EC_50_‐concentrations (Table 1).

Interestingly, for estriol, hydrocortisone, and aldosterone, which showed higher EC_50_ values, we observed a different binding mode (Figure 3C). In fact, these compounds cannot assume the binding mode of 17α‐hydroxyprogesterone, because in that conformation they would make clashes with Trp88 (specifically, clashes would involve ring A of the hydrophobic core of estriol, and the methyl in position 10, between rings A and B of hydrocortisone and aldosterone, see Figure S2).

For TAS2R14, interestingly, the most active compound (estrone) has an aromatic ring as ring A. Within the receptor binding site, the hydrophobic core is predicted to accommodate the center of the cavity, pointing the polar features toward transmembrane domain (TM) 2 and TM5 to establish two H‐bonds with Ser65 and Thr184, respectively. Estradiol establishes a very similar binding pose (Figure 3D). The predicted poses of compounds that have a nonaromatic ring A were generated based on the established pose of cholesterol in the experimental structure (Figure 3E). Therefore, we identified two different binding modes for compounds with aromatic and nonaromatic moieties in ring A (Figure 3F).

## 4 Discussion

In the present study, it was demonstrated that all steroid hormones and other tested compounds activated one of the two or both bitter taste receptors, TAS2R14 and TAS2R46. Among the two receptors, the TAS2R46 showed, in most cases, elevated sensitivity and resulted in stronger signals. In fact, the threshold concentrations of 10 nM for 17α‐hydroxyprogesterone, deoxycorticosterone, and epitestosterone when stimulating the TAS2R46 are, at present, among the lowest activation concentrations ever reported for any human and nonhuman bitter taste receptor.

Apart from the high sensitivity of, in particular, the TAS2R46, the data demonstrated an astonishing level of selectivity for both receptors. The fact that one of the three compounds showing the highest potency for the TAS2R46, the 17α‐hydroxyprogesterone, exhibits no activation of the TAS2R14, whereas the single most potent agonist of the TAS2R14, estrone, elicits no responses of TAS2R46 expressing cells, suggests functional specialization. Whereas estrone is one of the three major endogenous estrogens, being weakly active as a female sex hormone [69], 17α‐hydroxyprogesterone is an endogenous progestogen steroid hormone, albeit weak compared to progesterone [70]. In both cases, small changes within the compounds are responsible for the observed dramatic changes in their activation properties. In the case of TAS2R14, the simple addition of a hydroxyl group to C17 of ring D of progesterone causes the loss of response; in the case of TAS2R46, reducing the oxo‐group at C17 of ring D of estrone to a hydroxyl group in estradiol results in a gain of function. Computational structure‐based investigations of steroid hormones to TAS2R14 and TAS2R46 orthosteric binding sites revealed that, despite the compounds sharing similar features (as shown by the pharmacophore analysis, Figure S1), they cannot occupy the binding site in the same manner (see Figure S3), thus explaining the differences in EC_50_ and receptor selectivity.

**FIGURE 4:**
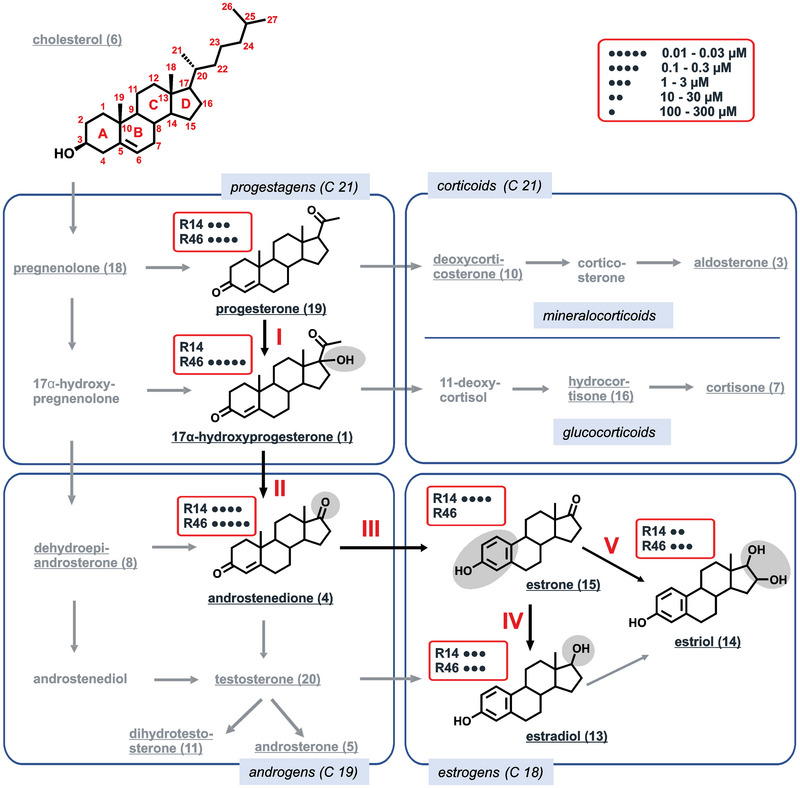
Important structural features for activation of bitter receptors TAS2R14 and TAS2R46 within biosynthesis pathways of steroid hormones [92]. The underscored names indicate the investigated metabolites. Black compound names indicate substantial changes in receptor activation profile. The shaded areas in the structures highlight structural differences from the respective precursor compound. **I**: Hydroxylation of progesteron [19] at C17 into 17α‐hydroxyprogesteron [1]. **II**: Deacetylation at C17 and oxidation of the hydroxyl of 17α‐hydroxyprogesteron [1] into androstenedion [4]. **III**: Aromatization of the A ring and demethylation at C10 of androstenedion [4] into estrone [15]. **IV**: Reduction of the carbonyl at C17 of estrone [15] into estradiol [13]. **V**: Hydroxylation at C16 and reduction at C17 of estrone [15] into estriol [14].

The structural changes occurring in the biosynthesis pathway of steroid hormones (Figure 4) clearly indicate that discrete conversion steps strongly impact TAS2R selectivity. Whereas TAS2R46 maintains its sensitivity during the conversion from progesterone to 17α‐hydroxyprogesterone and further to androstenedione at a rather constant high level, TAS2R14, which is initially quite sensitive for progesterone, loses its responsiveness upon hydroxylation of progesterone at C17 completely and regains activation by conversion from 17α‐hydroxyprogesterone to the androgen androstenedione. Hence, TAS2R14 could serve as an off‐switch for biosynthetic intermediates with reduced steroid hormone activity. A similar switch is observed for TAS2R46, which loses its responsiveness upon aromatization of ring A and demethylation at C10 during the conversion of androstenedione to estrone. Estrone is a weak estrogen and intermediate of the powerful estrogens, estriol and estradiol. For both compounds, TAS2R46 regains responsiveness. The expression patterns of both receptors would be in agreement with such hypothetical roles in regulatory processes in steroid hormone synthetic pathways (cf https://www.proteinatlas.org/search/TAS2R): In general, TAS2R14 mRNA is much more abundant than the TAS2R46 mRNA. Apart from the cerebellum, pituitary, and urinary bladder, TAS2R46 mRNA was detected at low levels in the ovary, cervix, and adipose tissue. In particular, the occurrence of TAS2R46 in adipose tissue is revealing, since this is, next to the ovary, where TAS2R46 is also found, the main tissue for the conversion of androstenedione to estrone and other estrogens [71]. This conversion is mediated by the enzyme aromatase. As mentioned above, the conversion of androstenedione to estrone would cause the shift from a high‐potency TAS2R46 ligand to a nonligand. This would be an attractive mechanism for a receptor involved in the metabolic control of this step, as it is activated by the educt and unresponsive to the product. Since the TAS2R46 is expressed in adipose tissue as well as in the ovary, and the main production site of estrogens changes in women from the ovary in premenopausal women to adipose tissue in postmenopausal women, TAS2R46 could control this step for a large part of women's lifetime [71]. TAS2R46 is not detectable in the adrenal glands (cf https://www.proteinatlas.org/search/TAS2R), and hence, despite its high sensitivity to progesterone and 17α‐hydroxyprogesterone, an involvement in a similar mechanism in this tissue is not envisaged. However, one could speculate that TAS2R14, which is highly sensitive to progesterone but insensitive to 17α‐hydroxyprogesterone, is involved in a similar regulatory event. The 17α‐hydroxylation of pregnenolone or progesterone irreversibly commits steroid production to cortisol/C19 steroid production and away from aldosterone synthesis [72]. Therefore, a bitter taste receptor responsive to the educt progesterone, but insensitive to the product 17α‐hydroxyprogesterone, could be involved in this regulation. Indeed, TAS2R14 is expressed in cells of the adrenal gland (cf https://www.proteinatlas.org/search/TAS2R), where the enzyme 17α‐hydroxylase cytochrome P450 is expressed, and this crucial branching in the steroid synthetic pathway occurs [72].

An important question with regard to the steroid hormone activation of human TAS2Rs is, whether these compounds trigger gustatory, nongustatory, or both types of responses. Regarding the taste response to steroid hormones, it is known that hydrocortisone, as well as prednisone, a synthetic glucocorticoid with similarity to hydrocortisone, indeed taste bitter [73, 74]. The superior sensitivity of TAS2R46 for hydrocortisone compared to TAS2R14 suggests that the taste response may arise from the activation of TAS2R46 or both responsive receptors. The general taste responsiveness of TAS2R46 is further confirmed by the bitter taste of orphenadrine, the active ingredient of the drug Norflex (https://pdf.hres.ca/dpd_pm/00015942.PDF). Since orphenadrine is a unique activator of TAS2R46 [41], no other TAS2R is likely to be responsible for the bitter taste of this compound, and hence, it would require further assumptions to claim that TAS2R46 recognizes orphenadrine, but not orally delivered steroid hormones. A similar argument can be made for the TAS2R14 and its unique activators noscapine, benzamide, and chlorhexidine [41], which have all been described as bitter [75, 76, 77].

One major source of animal‐derived steroid hormones could be cow's milk. However, the rather low levels of estrone (0.13 µg), estradiol (0.02 µg), and progesterone (10 µg) per liter milk [78] are neither taste‐relevant, nor do they contribute substantially to the daily endogenous production (♂ 420 µg progesterone, 25 µg estrogen; ♀19,600 µg progesterone, 100 µg estrogen [78]) and, consequently, circulating levels of these hormones. In stark contrast to these, the phytoestrogen genistein is found at much higher levels in food products, for example, the fermented “tempe” made from soy beans can contain up to 18.7 mg/100 g (∼0.7 mM) [79], which is well above the threshold concentration for the activation of TAS2R14 (10 µM). This may not only account, at least in part, for the well‐documented bitterness of “tempe” [80], but it also indicates the possibility that overconsumption indeed could bear an elevated risk for hormonal activities. Similar to the newly discovered negative consequences of anabolic steroid misuse, such as reduced male fertility [81], chronic overingestion of phytoestrogens could result in pathologies, some of which might be due to the activation of endogenous bitter taste receptors.

It seems to be common knowledge that during pregnancy, the female taste perception undergoes pronounced changes [82]. Although this observation is only supported by some studies, whereas other studies did not find a correlation, pronounced hormonal changes are indisputable. In particular, the levels of progesterone exhibit enormous surges of more than 100‐fold in the course of pregnancy [83]. It is tempting to speculate that such substantial changes could result in gustatory and taste receptor‐mediated physiological changes as well.

Already, Kim and colleagues reported the existence of a truncated variant of the TAS2R46 gene with an allele frequency of the mutated allele of 24% [13]. The lack of function was confirmed by Roudnitzky et al. [84]. With a frequency of 8% of the human population being homozygous carriers of the truncated TAS2R46 gene [84], we assume that phenotypical manifestations should be rather abundant. As the response of TAS2R46 to low concentrations of steroids due to its far superior sensitivity cannot be compensated by other TAS2Rs, including TAS2R14, a careful study of genotype−phenotype correlations should be attempted in the future.

There are several steroid hormones present in human blood at concentrations exceeding the threshold concentrations of TAS2R14 and/or TAS2R46. One of these is progesterone, which, during pregnancy, increases from levels well below the threshold concentration of TAS2R46 (0.03 vs. 0.002 µM) to 0.493 µM [66], a strongly activating concentration for this receptor. In male individuals, testosterone concentrations in blood can reach the threshold of TAS2R46 [66], a fact that suggests that the concentration at the production site, for example, testicles, certainly exceeds the threshold concentration.

Most intriguingly, hydrocortisone can reach under conditions of stress concentrations of 8.69 µM [66], which is already close to the half‐maximal activating concentrations for the TAS2R46 and hence should elicit a bitter percept. Indeed, it was shown that human individuals ranked the bitterness of a saccharin solution under stress conditions as more bitter, whereas the sweetness perception was unchanged [85]. It is tempting to speculate that part of the explanation for the elevated bitterness arises from the elevated secretion of hydrocortisone‐enriched saliva during the tasting of the saccharine. If true, one needs to conclude, first, that stress can be “tasted,” a phenomenon in agreement with reports on chemosensory aberrations occurring during stress [86], and second, that chronic stress should negatively impact the function of the TAS2R46 due to receptor desensitization. This would fit an observation in stressed mice, which exhibit reduced taste responses and show a downregulation of taste receptor genes, including bitter taste receptors [87]. Therefore, we would speculate that the desensitization of stress hormone‐responsive bitter taste receptors may add to the reduced taste perception. This bears multiple important implications. As bitter taste receptors are expressed in myocytes of the heart and bitter stimulation results in negative inotropy [32, 88], heart failure may occur more frequently during phases of stress. Bitter taste receptors are abundantly expressed in spermatozoids, and it has been documented that the number of sperm cells as well as their quality is affected by stress [89]. Moreover, stress is known to induce heartburn through overproduction of stomach acid [90], which could be related to bitter taste receptors in the stomach [91] and their activation by stress hormones. Another corticoid hormone reaching levels above or at the threshold concentration for both receptors, TAS2R14 and TAS2R46, is deoxycorticosterone in amniotic fluid [66]. Whether this high level of the hormone indicates an explicit function for fetal development or not is not clear. Dehydroepiandrosterone sulfate levels in blood [66] are clearly above the threshold concentration for the activation of TAS2R46. This hormone serves as a buffering hormone for male and female sex hormones. In this case, not the activation of TAS2Rs but the lack of activation should serve as a trigger, for example, the production of dehydroepiandrosterone in the cortex. While also androsterone is present at the necessary threshold concentration [66] for the activation of the TAS2R46, it will hardly result in more than chronically low stimulation of the receptor.

## 5 Conclusion

In summary, the finding that steroid hormones represent very potent activators of human TAS2Rs adds to our understanding of the intricate complexity of bitter taste receptor functions and paves the way for more clinical investigations. Whether steroid hormones may actually represent bona fide tastants or rather mostly endogenous agonists or both, remains to be seen in the future; however, there are good reasons to respond to (and reject) exogenous steroid hormones because of their profound pharmacological activities, as well as to align our physiology to circulating levels of them.

## Author Contributions

Conceptualization: Maik Behrens; Formal analysis and investigation: Maik Behrens, Tatjana Lang, Francesco Ferri, Florian Ziegler, and Antonella Di Pizio; Writing—original draft preparation: Maik Behrens and Tatjana Lang; Writing—review and editing: Maik Behrens, Tatjana Lang, and Antonella Di Pizio; Supervision: Maik Behrens and Antonella Di Pizio.

## Conflicts of Interest

The authors declare that they have no competing interests.

## Funding

This research did not receive external funding.

## Supporting information




**Supplementary Materials**: nyas70172‐sup‐0001‐SuppMat.docx

## Data Availability

All data generated or analyzed during this study are included in this published article (and its Supplementary Information Files).
