# Supplementary material for: Steroid Hormones Are Potent and Putatively Endogenous Activators of Human Bitter Taste Receptors
Source: Ann N Y Acad Sci. 2026 Jan 2;1556(1):e70172. doi: 10.1111/nyas.70172 (PMC12915477; doi:10.1111/nyas.70172)
Supplement: Supplementary file 1 — Supplementary Materials: nyas70172‐sup‐0001‐SuppMat.docx [file NYAS-1556-0-s001.docx]

Supplementary information

**Steroid hormones are potent and putatively endogenous activators of human bitter taste receptors**

Tatjana Lang^1^, Francesco Ferri^1,2^, Florian Ziegler^1^, Antonella Di Pizio^1,2^, and Maik Behrens^1,*^

^1^Leibniz Institute for Food Systems Biology at the Technical University of Munich, Freising, Germany; ^2^Professorship for Chemoinformatics and Protein Modeling, Technical University of Munich, Freising, Germany

**Supplementary table 1.** Steroid hormones, cholesterol, genistein, and stigmasterol used for bitter taste receptor screening and characterization. The maximally applied concentrations in µM (C_max_), CAS-numbers (CAS-no.), % purities, and vendors are provided.

| no. | compound | C_max_ | CAS-no. | %-purity | from |
| --- | --- | --- | --- | --- | --- |
| 1 | 17α-Hydroxyprogesterone | 100 | 68-96-2 | 99.5 | Dr. Ehrenstorfer GmbH |
| 2 | Adrenosterone | 300 | 382-45-6 | 98 | Aldrich |
| 3 | Aldosterone | 300 | 52-39-1 | 98 | Acros organics |
| 4 | Androstenedione | 100 | 63-05-8 | 98 | Sigma-Aldrich |
| 5 | Androsterone | 100 | 53-4-8 | 99 | Sigma |
| 6 | Cholesterol | 100 | 57-88-5 | 99 | Sigma-Aldrich |
| 7 | Cortisone | 300 | 53-06-5 | 98 | Sigma |
| 8 | Dehydroepiandrosterone | 100 | 53-43-0 | 99 | Acros organics |
| 9 | Dehydroepiandrosterone sulfate | 100 | 78590-17-7 | 93 | Sigma-Aldrich |
| 10 | Deoxycorticosterone | 100 | 68-96-2 | 97 | Sigma |
| 11 | Dihydrotestosterone | 100 | 521-18-6 | 99 | Sigma |
| 12 | Epitestosterone | 300 | 481-30-1 | 99 | Sigma-Aldrich |
| 13 | Estradiol | 100 | 50-27-1 | 99 | Alfa Aesar |
| 14 | Estriol | 100 | 50-27-1 | 97 | Sigma-Aldrich |
| 15 | Estrone | 100 | 53-16-7 | 99 | Acros organics |
| 16 | Hydrocortisone | 100 | 50-23-7 | 98 | Aldrich |
| 17 | Methyltestosterone | 300 | 58-18-4 | 97 | Sigma-Aldrich |
| 18 | Pregnenolone | 100 | 145-13-1 | 99 | Acros organics |
| 19 | Progesterone | 300 | 57-83-0 | 99 | Sigma-Aldrich |
| 20 | Testosterone | 300 | 58-22-0 | 99 | Sigma |
| 21 | Genistein | 100 | 446-72-0 | 99 | Alfa Aesar |
| 22 | Stigmasterol | 100 | 83-48-7 | 95 | Sigma-Aldrich |

**Figure S1**


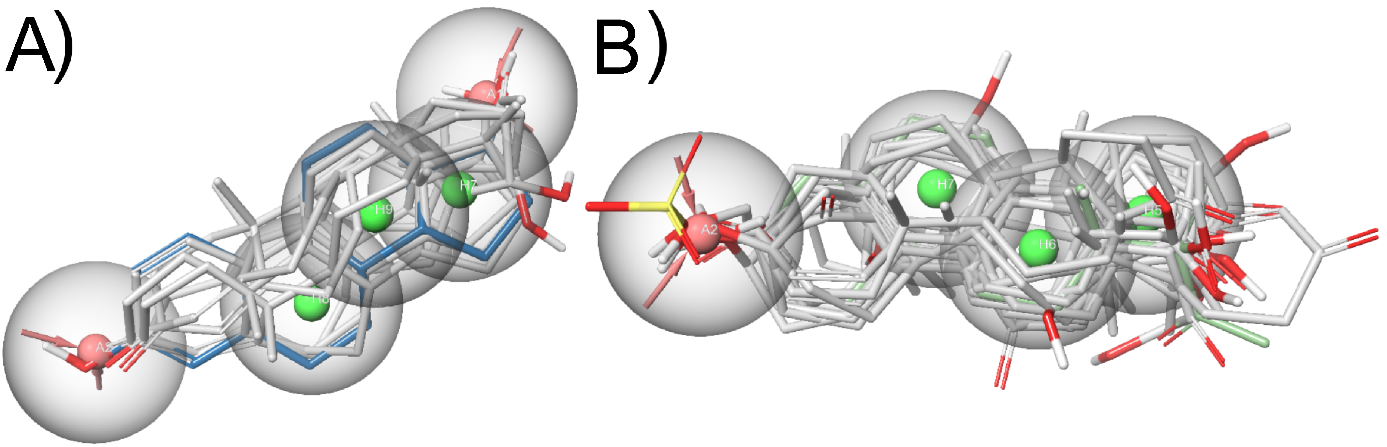


**Figure S1.** Pharmacophore models obtained from identified agonists for A) TAS2R14 (reference ligand in blue) and B) TAS2R46 (reference ligand in green).

The TAS2R46 model is made of four features: three hydrophobic features matching ring B, C, and D in the hydrophobic core, and one acceptor feature matching the hydroxyl/carbonyl oxygen on ring A. The TAS2R14 model is instead made of five features. With respect to the TAS2R46 model, it has an additional acceptor feature matching the hydroxyl/carbonyl oxygen on ring D. The additional feature can be due to the fact that, with fewer compounds, the alignment allows for an increased number of features matched. Therefore, in terms of features, the compounds that activate TAS2R46 and those that activate TAS2R14 are not different. To investigate the structure-activity relationships of the two receptor agonist profiles, we proceed with structure-based investigations to understand how, in the receptor binding site, these similar molecular features could elicit activity and receptor selectivity.

**Figure S2**


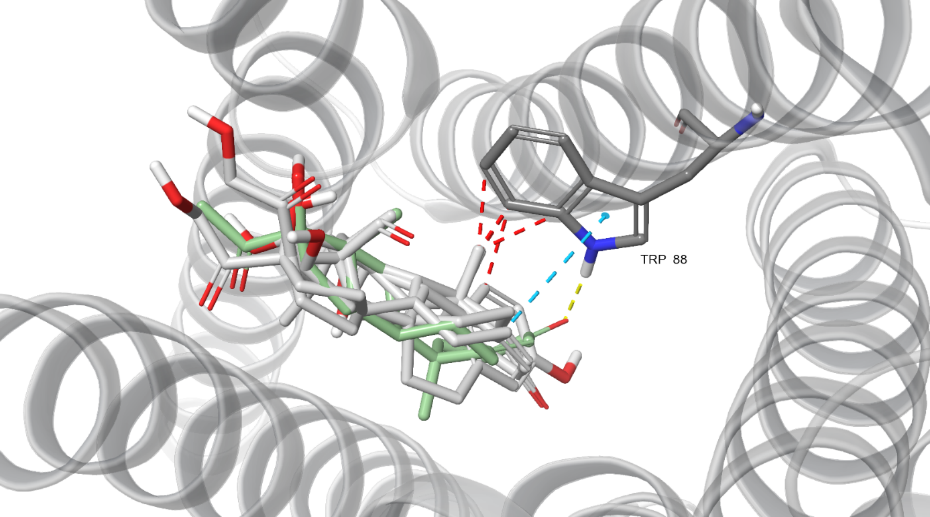


**Figure S2.** Alignment of estriol, hydrocortisone, and aldosterone (white) on 17α-hydroxyprogesterone showing multiple clashes (red dashed lines) between Trp88 and methyl groups (hydrocortisone and aldosterone) and aromatic ring (estriol) in TAS2R46.

**Figure S3**


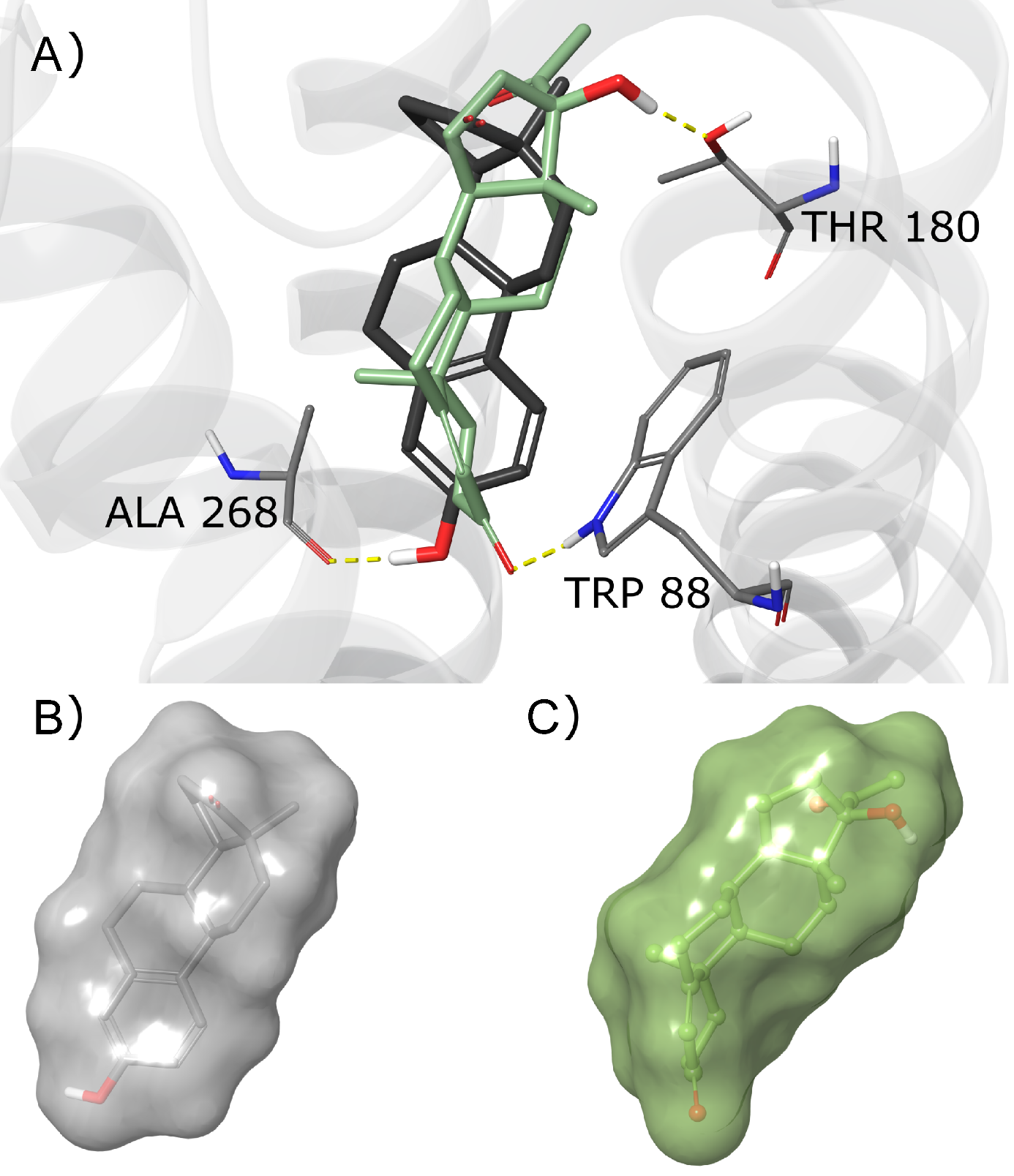


**Figure S3.** A) Docking poses of 17α-hydroxyprogesterone and estrone on TAS2R46; B) Surface of the docked estrone molecule (gray); C) Surface of the docked 17α-hydroxyprogesterone (green).

Direct comparison of the docking poses of compound 1 (17α-hydroxyprogesterone, shown in green) and compound 15 (estrone, shown in black). As illustrated, compound 15 should not establish the same key molecular interactions with TAS2R46 as reference compound 1. While compound 1 is predicted to form hydrogen bonds with the sidechains in the pocket, specifically TRP88, compound 15 is only predicted to have a hydrogen bond with the backbone. Moreover, the model highlights that compound 15 is bulkier in its overall structure (Fig. S3B-C, compound 15 in gray and compound 1 in green). This steric hindrance prevents the ligand from fitting optimally into the TAS2R46 binding site, which could explain its lack of TAS2R46 activation, thereby contributing to the observed selectivity.
